# Experience with exchange and archiving of raw data: comparison of data from two diffractometers and four software packages on a series of lysozyme crystals

**DOI:** 10.1107/S0021889812044172

**Published:** 2012-12-08

**Authors:** Simon W. M. Tanley, Antoine M. M. Schreurs, John R. Helliwell, Loes M. J. Kroon-Batenburg

**Affiliations:** a School of Chemistry, Faculty of Engineering and Physical Sciences, University of Manchester, Brusnwick Street, Manchester M14 9PT, UK; b Crystal and Structural Chemistry, Bijvoet Center for Biomolecular Research, Utrecht University, Padualaan 8, Utrecht, 3584 CH, The Netherlands

**Keywords:** data exchange, data archiving, metadata

## Abstract

The International Union of Crystallography has for many years been advocating archiving of raw data to accompany structural papers. Recently, it initiated the formation of the Diffraction Data Deposition Working Group with the aim of developing standards for the representation of these data. A means of studying this issue is to submit exemplar publications with associated raw data and metadata. A recent study on the effects of dimethyl sulfoxide on the binding of cisplatin and carboplatin to histidine in 11 different lysozyme crystals from two diffractometers led to an investigation of the possible effects of the equipment and X-ray diffraction data processing software on the calculated occupancies and *B* factors of the bound Pt compounds. 35.3 Gb of data were transferred from Manchester to Utrecht to be processed with *EVAL*. A systematic comparison shows that the largest differences in the occupancies and *B* factors of the bound Pt compounds are due to the software, but the equipment also has a noticeable effect. A detailed description of and discussion on the availability of metadata is given. By making these raw diffraction data sets available *via* a local depository, it is possible for the diffraction community to make their own evaluation as they may wish.

## Abstract 2

A systematic analysis of diffraction data of 11 different lysozyme crystals (used for cisplatin and carboplatin binding studies), obtained with four diffraction data processing software packages and from two different diffractometers, serves as a pilot study for archiving raw diffraction data and associated metadata. The availability of the raw diffraction images allows for independent assessment of software packages.

## 1. Introduction   

There is increasing interest in depositing or archiving raw data of scattering experiments with publication of structural papers. This interest in archiving raw data is common to all scientific fields, as highlighted in the ICSU SSCID Report (2011 ▶). International Union of Crystallography (IUCr) journals are leaders in the archiving of derived and processed data with crystal structure papers either with articles in *Acta Crystallographica Sections B*, *C* and *E* or in close linking with the PDB (Protein Data Bank; Berman *et al.*, 2000 ▶) in the case of *Acta Crystallographica Sections D* and *F*. Detailed consideration is now being given to the benefits, and extra costs, of extending the data archiving paradigm to also now include raw data such as diffraction data images. The Diffraction Data Deposition Working Group has been set up by the IUCr, and a variety of reports and ongoing community feedback can be found at the IUCr forum devoted to this matter. Reasons for archiving raw data include to improve the record of science, to ensure the reproducibility and allow detailed checks of scientific data, to safeguard against fraud, and to allow reanalysis with future improved techniques. The digital object identifier for each data set underpinning a published paper at an archive local to where the data were measured is a plausible model to move these developments forward; at the University of Manchester this is being launched in September 2012 and will be available in 2013. As an interim measure a link is provided to a personally maintained web site (http://rawdata.chem.uu.nl). Cost requirements of the long-term professional stewardship of digital data storage and large bandwidth access are important issues, but a further requirement is the provision for a sufficient level of metadata, to allow future use of the data. This paper addresses the challenges and possibilities of exchanging raw data with associated metadata for data processing with non-native software, *i.e.* not associated directly with a given piece of measuring equipment, for example, from a given manufacturer.

In protein crystallography, X-ray diffraction data are often obtained from synchrotron beamlines that provide high-brilliance beams and rapid data collections. While the synchrotron installations gradually gained in performance, the development of home sources also continued. Currently, microfocus X-ray sources with matching multilayer optics and high-performance detectors are available and can compete with second-generation synchrotron beamlines. They can also be technically appropriate and indeed, being local, highly convenient. Manufacturers of diffraction equipment for applications in macromolecular and chemical crystallography provide integrated software for designing data collection strategies and data processing. The end result of a diffraction experiment is a series of recorded diffraction data images. Metadata are contained in the headers of these image files or internally on a server computer. The manufacturer’s internal software usually takes care of necessary corrections, such as for detector non-uniformities by using a flood-field image or for distortion due to the fibre optic taper in CCDs. Detaching images from the server computer for processing with ‘alien software’ requires finding all necessary metadata. Several independent data processing software packages have been developed for protein crystallography during the past two to three decades, *e.g. MADNES* (Messerschmidt, 1986 ▶; Messerschmidt & Pflugrath, 1987 ▶), *DENZO*/*HKL-2000* (Otwinowski & Minor, 1997 ▶), *Mosflm* (Leslie, 1999 ▶), *XDS* (Kabsch, 1988 ▶) and, most recently, *EVAL* (Schreurs *et al.*, 2010 ▶). The first three have been largely optimized for synchrotron data and later adapted to read several commercial detector formats, and (partially) also the possible goniometer geometries. *EVAL*, by contrast, was developed primarily for use with Nonius equipment with a four-circle Kappa goniometer, while specific efforts were made to implement a large range of detector formats and goniometer geometries.

In a preceding paper (Tanley *et al.*, 2012 ▶) the binding of cisplatin or carboplatin to histidine in a protein (lysozyme) was described, based on diffraction data of 11 crystals of different chemical compositions. These were measured on two different X-ray diffractometers, namely a Rigaku Micromax-007 generator with Cu rotating anode equipped with an R-AXIS IV image plate and Osmic confocal mirrors, and a Bruker MICROSTAR Cu rotating anode equipped with a CCD ‘Pt detector’ and HELIOS confocal optics (X8 PROTEUM2). Initially, data processing was done with the equipment’s internal software. After protein model refinement of the 11 crystal structures, the average isotropic atomic *B* factors seemed to vary systematically between the two diffractometers, suggesting a systematic deviation in the treatment of weak intensity reflections. Since the scientific goal was to determine metal-binding occupancies, a possible influence of systematic errors in the *B* factors needed to be ruled out and/or corrected for. This led to a joint undertaking of the Utrecht and Manchester structural chemistry research groups to yield processed quality data from just one suite of software, *EVAL*. The X-ray diffraction data obtained from the 11 lysozyme crystals had been measured using the two diffractometers and processed with four software packages (Tanley *et al.*, 2012 ▶). Most of the crystals diffracted to 1.7 Å. In total the diffraction data images (the ‘raw’ data) transferred between Manchester and Utrecht occupied 35.3 Gb of disk space and the process took a few days. In this paper we will address the availability and use of metadata, specifically in the case of processing with *EVAL*. The results and observations obtained allow detailed informed comment on the archiving of such raw diffraction data and their reprocessing after and beyond their initial study with a variety of software programs.

## 2. Materials and methods   

The 11 lysozyme crystals (hen egg white lysozyme, HEWL) co-crystallized with cisplatin or carboplatin, with dimethyl sulfoxide or under aqueous conditions including *N*-acetyl­glucos­amine, along with different cryosolvents, were prepared as described in the previous paper (Tanley *et al.*, 2012 ▶). Out of the 11 crystals studied, six (labelled 1–4, 10 and 11) were collected on a Rigaku R-Axis IV image plate and the remaining five (5–9) were collected on a Bruker PLATINUM 135 CCD detector, both using an X-ray wavelength of 1.5418 Å and at a temperature at the crystal sample set to 100 K. The data collection strategies (Table 1 ▶) were automatically chosen by the integrated strategy software *PROTEUM2* on the Bruker equipment, and data were collected simply by a 360° ϕ scan on the R-AXIS, while requiring a redundancy of at least 8.0.

## 3. Results   

Each sequence of X-ray diffraction data images was processed with three different packages, namely the equipment’s software [either d*Trek (Pflugrath, 1999 ▶) or *PROTEUM2* (Bruker, 2006 ▶)], *Mosflm* (Leslie, 1999 ▶) [using *SCALA* (Evans, 2006 ▶) for scaling] and *EVAL* [using *SADABS* (Sheldrick, 1996 ▶) for scaling]. Tables 2 ▶ and 3 ▶ list the diffraction data reflection intensity integration and the subsequent protein model refinement statistics. In the cases where the processed X-ray data and three-dimensional coordinate sets were deposited with the PDB, a PDB code is given.

### 3.1. Comparison of hardware   

#### 3.1.1. Image formats and image headers   

**Figure 1:**
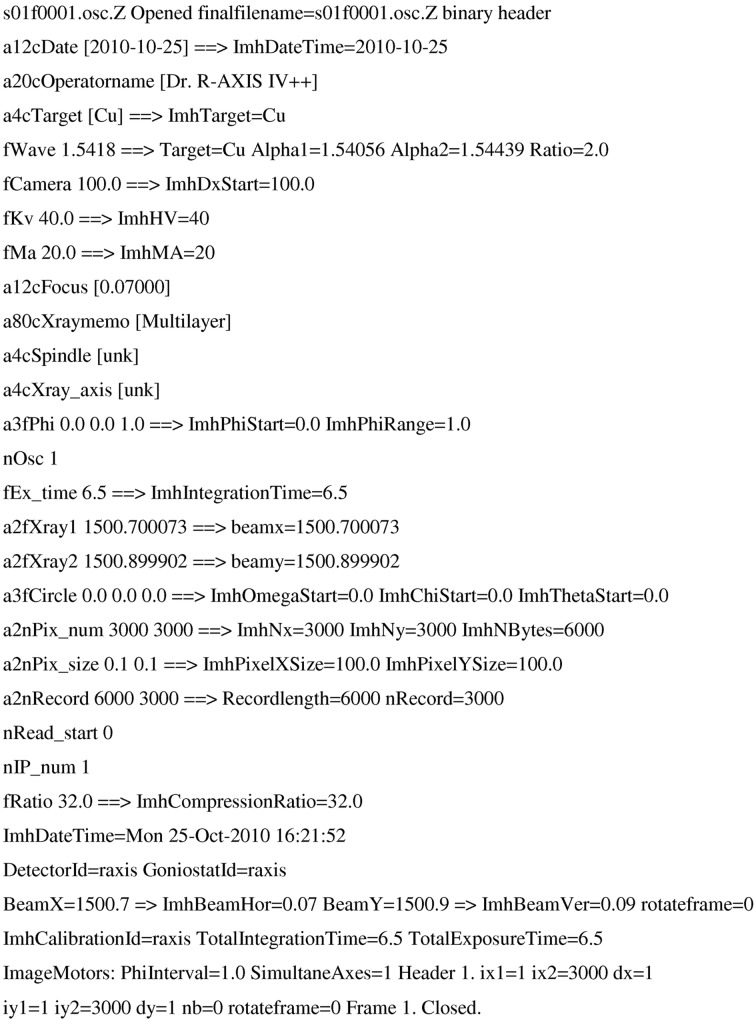
Part of an R-AXIS IV diffraction data image header.

An example of an R-AXIS IV image plate header (Fig. 1 ▶) shows the information *EVAL* extracts. After the arrow, *EVAL*’s interpretation is given. The starting and ending rotation angles of the spindle axis were read (a3fPhi 0.0 0.0 1.0), as well as the goniometer starting angles (a3fCircle 0.0 0.0 0.0, for ω, χ and swing θ); the rotation direction was known to us *a priori* (see next section). The number of pixels (3000 × 3000) and pixel size (100 µm) for each diffraction image were read. The direct beam position on the detector is indicated by the beam centre positions a2fXray1 and a2fXray2 in pixels. The R-AXIS has two image plates, allowing a frame to be read while the next image plate is exposed (nIP_num). The parameter ImhCompression refers to the way unsigned short-integer pixel values larger than 32 768, so-called overflow pixels, should be interpreted. No distortion or non-uniformity corrections are needed. Every diffraction image takes 18 Mb of disk space. When the images were received in Utrecht after network transfer from Manchester they were immediately compressed, using *compress* (from the *ncompress* package), to 3.8 Mb. *ncompress* is public domain software that uses the LZW (Lempel–Ziv–Welch) algorithm for lossless data compression (Welch, 1984 ▶). *EVAL* uses *compress −d* to unpack the images on the fly.

**Figure 2:**
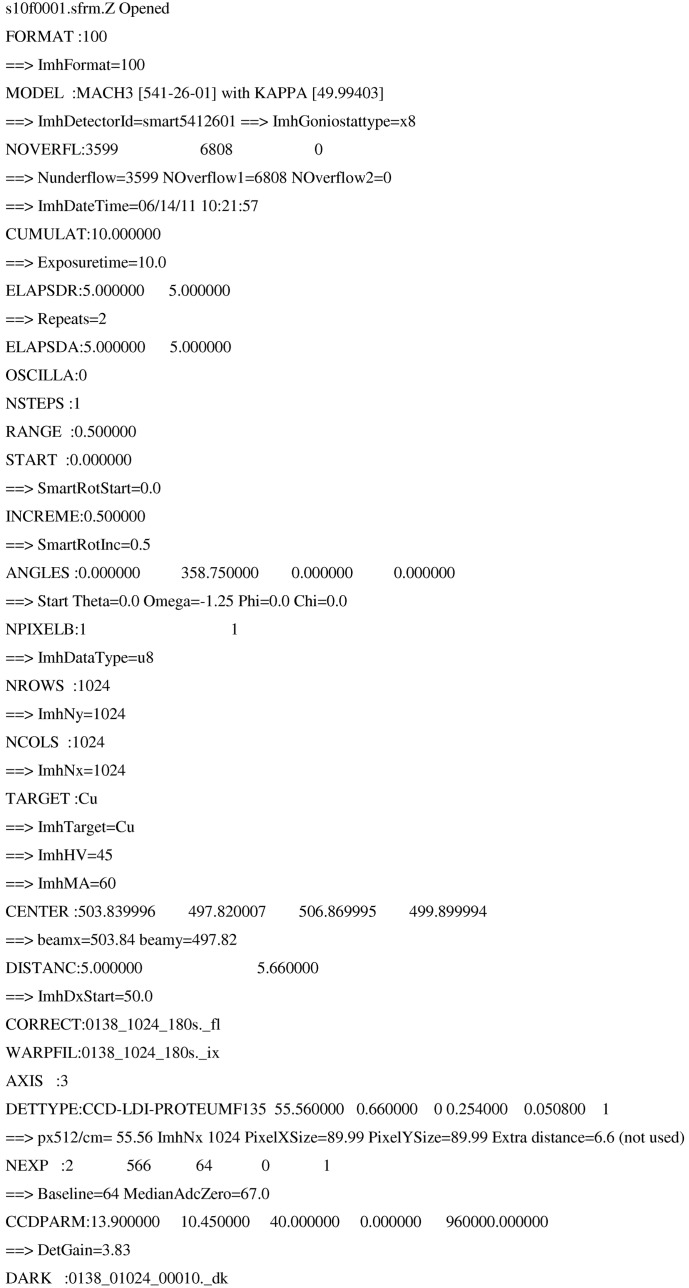
Part of a Bruker diffraction data image header.

The header information of a Bruker PLATINUM CCD detector diffraction data image (Bruker format) is shown in Fig. 2 ▶. The starting angles (ANGLES: 0.0 358.75 0.0 0.0) of the goniometer in Eulerian space (swing 2θ, ω, ϕ and χ) are given and later in the header the crystal sample rotation axis is defined, ϕ in this case (AXIS: 3). Pixel intensities are stored as 1 byte integers (NPIXELB = 1), and if the number is 255, additional bytes will follow at the end of the image in an overflow table. If not too many overflows occur this is a very efficient format. The baseline offset (from NEXP:), which is needed to store negative numbers in 1 byte integers, has to be subtracted. The gain of the detector is derived from the numbers after the keyword CCDPARM. The detector has 1024 × 1024 pixels (binned mode) and the pixel size is 89.99 µm. The detector position in pixels is given by CENTER. The non-uniformity of the detector is normally corrected by the manufacturer with a flood-field image (CORRECT:0138_1024_180s._fl). The spatial distortion information is contained in a so-called .p4p or spin file, but in the absence of a conversion script to make a distortion polynomial for *EVAL* (as was delivered by Bruker), the images should be ‘unwarped’ (WARFIL:0138_1024_180s._ix) before being processed. The latter was done for the *Mosflm* integrations. The Bruker software corrects the CCD images for dark current, *i.e.* ‘signal without X-rays’ built up in the detector electronics for the given measurement time (DARK: 0138_01024_00010._dk). In *EVAL* the Kappa goniometer option is fully implemented, whereas it is not in *Mosflm*. We had to resort to integration of each scan (rows in Table 1 ▶) separately, and scaled them later with *SCALA*. Each image takes about 1 Mb of disk space, and 800 kb when compressed, which indicates that the Bruker format is very space efficient.

Diffraction data image formats were kindly provided by Rigaku and Bruker during the development of *EVAL* in previous years. There is no way to extract data from the image file unless these formats are known. Even once this information is available, header information is often not comprehensive and unambiguous.

#### 3.1.2. Metadata   

The image format of the Rigaku image plate contains a binary header that did not provide all the information needed. In fact the layout of the goniometer axes and the sense of the crystal rotation axis can be one of the most laborious problems to deal with when implementing data processing for an unknown goniometer. A field in the header for the orientation of the spindle axis is reserved but did not contain a value in the current data (a4cSpindle in Fig. 1 ▶). Also the fastest and slowest running coordinates of the pixel data are not given. Assuming the spindle is perpendicular to the X-ray beam, it is obvious what the sense of rotation is, *i.e.* clockwise or anticlockwise, by looking at a few consecutive frames. However, looking at the diffraction image means that the horizontal and vertical axes and their direction on the detector are already interpreted (in fact there are eight possible ways of doing so). A consistent interpretation was found previously, also helped by the visibility of the beam stop and cryo nozzle, and used in the current work. We therefore had prior knowledge on how to interpret the data. Rigaku Corporation has developed a new ASCII header type that contains all the definitions for orientations of goniometer axes and for the detector axes in the laboratory frame, so that a comprehensive set of metadata is then provided.

The Bruker image format contains the model of the goniometer and the fixed κ angle (MACH3 and KAPPA in Fig. 2 ▶) and the goniometer rotation angles defined as Euler angles. Again we learned from previous data that the rotation directions for 2θ, ω and χ are opposite to that of ϕ. The Bruker format potentially gives refined detector positions in terms of pitch, roll and yaw, but these are ignored by *EVAL*, as these will be the result of the *PEAKREF* (Schreurs, 1999 ▶) refinement.

Authors of integration software such as *DENZO*, *Mosflm*, *MADNES*/d*Trek and *XDS* have done the same tedious unravelling of detector formats. To avoid having to go through such efforts the CBF/imgCIF format was developed (Bernstein & Hammersley, 2005 ▶; Bernstein, 2005 ▶). It provides a structure in which all metadata can be found in one place. It consists of an ASCII imgCIF header and binary (CBF) or ASCII-based encoded data blocks. The binary format is space efficient owing to the use of compression algorithms, like Byte_offset compression, and is useful for large images and data transfer between collaborating groups, exactly the situation we were engaged with. Three categories of data exist – ARRAY data, AXIS data and DIFFRN data – allowing a unique definition of how to interpret the data, and no prior knowledge would be required if all data items were filled in. This is often not the case, however; *e.g.* PILATUS detector image files contain all relevant metadata in just a small comment line block, the so-called mini CBF format.

#### 3.1.3. Data processing   

The strategies chosen in *CrystalClear* (Rigaku Corporation, Tokyo, Japan) and with the *PROTEUM2* processing software were rather different. The Rigaku equipment has only a single spindle axis ϕ and the total rotation range was simply either 180 or 360°, in each case with 1° per frame, the detector being positioned at a distance to exploit the maximum diffraction resolution the crystal offered. The X-ray generator was set to 20 mA and exposure times varied between 3 and 10 min. Image plates have a relatively low quantum gain compared with a CCD (although their performance is very good compared with the photographic X-ray film of yesteryear) and therefore need correspondingly longer exposure times, approximately a factor of 2–3; however, they have a superb uniformity, a very large dynamic range and no spatial distortion. The Bruker X8 PROTEUM2 CCD diffractometer has a smaller detector aperture but can be rotated in the normal plane: and indeed this option was exercised and detector swing angles used in these data collections varied between −15 and 20°. The CCD has a better quantum gain but uses a fibre optic taper, causing non-uniformity and spatial distortion. Furthermore, it is equipped with a Kappa goniometer, allowing scans around ω and ϕ at different κ positions. The X-ray generator was set to 60 mA and exposure times were 10–30 s per 0.5°. The difference between the measuring strategies on the two apparatuses did not have a large effect on the completeness or the redundancies (although the total measuring time was shorter on the PROTEUM2 than on the R-AXIS IV); the data redundancies varied between 12.1 and 25.3 for Rigaku and 14.6 and 31.4 for Bruker (*EVAL* data in Tables 2 ▶ and 3 ▶).

**Figure 3:**
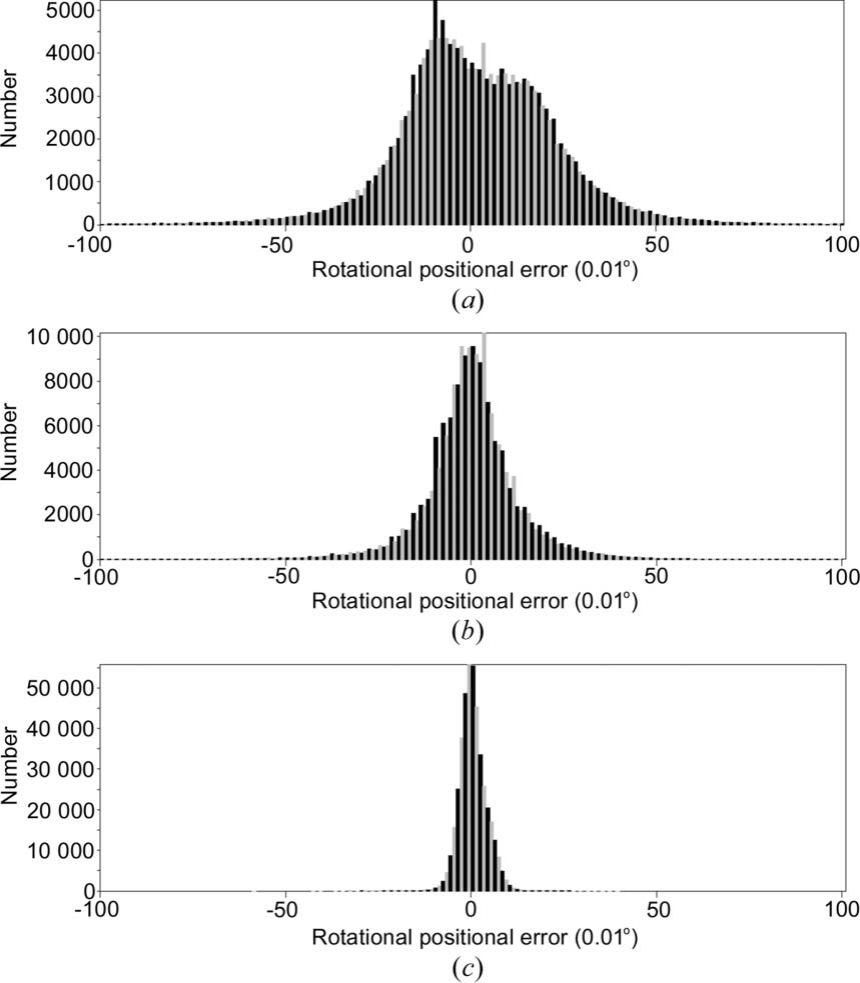
Rotational positional errors (in units of 0.01°) in Rigaku data set 1 (4dd0) (*a*) with a single unit-cell orientation matrix and (*b*) with different orientation matrices for each box file. (*c*) Bruker data set 5 (4dd1).

We will focus on the *EVAL* results when comparing the two diffractometers as this has the overriding advantage, since identical processing software is used for the two devices, of giving a consistency of treatment of the diffraction data images. Indexing of all diffraction data was straightforward with *DIRAX* (Duisenberg, 1992 ▶), except for 4dd1 and 4ddc (see later). Each of the crystal unit-cell dimensions and orientation matrix were written to a .rmat file, which the program *VIEW* (Schreurs, 1998 ▶) uses to predict reflection positions and to write corresponding reflection boxes for integration within *EVAL15*. After *EVAL15* processing, a post refinement with *PEAKREF* gave the final unit cells as listed in Tables 2 ▶ and 3 ▶. Any disagreement between predicted and refined reflection positions could be a reason to repeat the *VIEW*/*EVAL15* cycle. Table 4 ▶ gives the final average errors in the reflected beam directions, in terms of reflection positions on the detector and in rotation angles when using a single unit-cell matrix. The Rigaku data needed refinement of unit-cell orientation for each box file to get acceptable agreement. The positional and angular average errors are still larger with these Rigaku data, typically 1–3 pixels and 0.1–0.2°. Though the latter is well within the rotation range of 1.0°, with the Bruker data it was possible to achieve both sub-pixel and sub-rotation range agreement. The post refinement was carried out by *PEAKREF*, a very flexible program that allows refinement of the unit-cell matrix, detector position, goniometer offsets and crystal position. No improvement of the predictions of the Rigaku diffraction spots could be obtained without releasing the orientation of the unit cell of each box file (a box file corresponds to a group of approximately 1000 reflections at roughly the same rotation angle). The improvement was mainly in the rotational positions, but no consistent interpretation in terms of crystal or detector movements or goniometer offsets could be found. Thus we introduced additional refinement of the orientation of the unit cell for each box file for the Rigaku data. Fig. 3 ▶ shows that the final agreement is comparable, given the difference in frame width, to the Bruker data. The case of the Bruker 4dd4 example is an exception. In this case apparently the crystal slips away from its original position as the orientation of the unit-cell axes was rotated approximately 3° between scans 2 and 3.

We cannot determine what causes the larger Rigaku data positional errors. The exchange of the two image plates after every exposure does not seem to affect their exact position, as no correlation with frame number, *i.e.* odd- and even-numbered images, was observed. The crystal orientation does not change in a systematic way as it is completely different for each experiment, so no correlation with the ϕ motor rotations was found either. We conclude that the crystal was not very well held to its initially placed position, possibly as a result of vibrating instrument parts.

#### 3.1.4. Standard deviations and statistics   

Every detector converts the X-ray photons that are absorbed by the phosphor layer into an electronic signal that is read out and stored in the image file. The detective quantum efficiency is a measure of the efficiency with which photons are detected and of the noise performance of the detector. It is defined as the signal-to-noise ratio of the output divided by that of the input. For an ideal detector this ratio would be 1.0. In practice a variety of factors reduce this number, like phosphor absorption efficiency, detector entrance window transmission, a phosphor noise factor, read-out noise, dark current and detector gain (Phillips *et al.*, 2002 ▶). An additional quantity of interest is the overall detector gain; we will define gain as the number of analogue-to-digital units (ADU) recorded per X-ray photon. Ideally a pixel intensity should be divided by the gain to obtain the X-ray photon counts, so that standard deviations of pixel intensities can be estimated using Poisson statistics. The R-AXIS header does not contain a value for the gain, so *EVAL* assumes it to be 1.0. In the Bruker header we found a gain value of 3.83 ADU per photon. Various published papers have shown that the standard deviations of diffraction intensities behave other than according to Poisson distributions; an early description can be found in the text book by Stout & Jensen (1968 ▶) and applies even to so-called photon-counting detectors. In the area detector ‘modern era’, Leslie (1999 ▶) and Popov & Bourenkov (2003 ▶) show that the variance of integrated intensities can be described by a second-order polynomial function in *I*: σ^2^ = *k*_0_ + *k*_1_ *I* + *k*_2_ *I* ^2^. The second term represents the error estimate from Poisson statistics (σ = *I* ^1/2^) corrected for the gain and Lorentz–polarization factor (Lp). The expression for σ^2^ can be rewritten as [(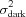 + 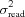) + (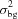 + *I*)] + (*g* *I*)^2^, where *I* is the net intensity, *g* is a factor to be determined during scaling, and the subscripts dark, read and bg denote the dark-current, read-out-noise and background contributions to σ. *EVAL* delivers standard deviations (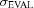) using the first two terms. Scaling programs like *SADABS* (Sheldrick, 1996 ▶) use an error model 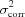 = *K*[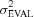 + (*g*〈*I*〉)^2^], in which *K* and *g* are refined, to achieve more reliable error estimates from internal standard deviations such that χ^2^ = 〈*N*∑(*I* − 〈*I*〉)^2^/(*N* − 1)σ^2^〉 is close to 1.0. The latter approach is also applied in *SCALA* (Evans, 2006 ▶). If the intensities *I* are on an absolute scale, *i.e.* represent actual X-ray photon counts, *SADABS* typically finds *K* values in the range 0.7–1.5 and *g* values in the range 0.02–0.04. An incorrectly estimated gain value will affect the estimated *I*/σ of reflections, but scaling programs will more or less correct for this, notably *via* the χ^2^ analysis. This correction may, however, not be in place when reflections are rejected on the basis of (*I* − 〈*I*〉)/σ > 4 (in the case of *SADABS*) and may lead to unwanted rejections. In *Mosflm*/*SCALA* the error model being used is sdFac[σ^2^ + sdBLp*I* + (sdAdd*I*)^2^]^1/2^ (as an example, for the processed diffraction data set of crystal 3, sdFac ≃ 1.5, sdAdd ≃ 0.02 and sdB = 3.17 for full reflections). d*Trek also uses a two-term adjustment of the standard deviations to match normal χ^2^ distributions. In a recent paper, Waterman & Evans (2010 ▶) showed that the standard deviations of intensities from profile fitting or summation integration are indeed underestimated and that simulation of the detection process, taking into account the various sources of error, leads to more realistic error estimates. Because of the similar procedures used for adjusting the standard uncertainties, we believe that comparison of *I*/σ values remains valid and is a necessary requirement of any physical science, of which crystal structure analysis is but one example.

**Figure 4:**
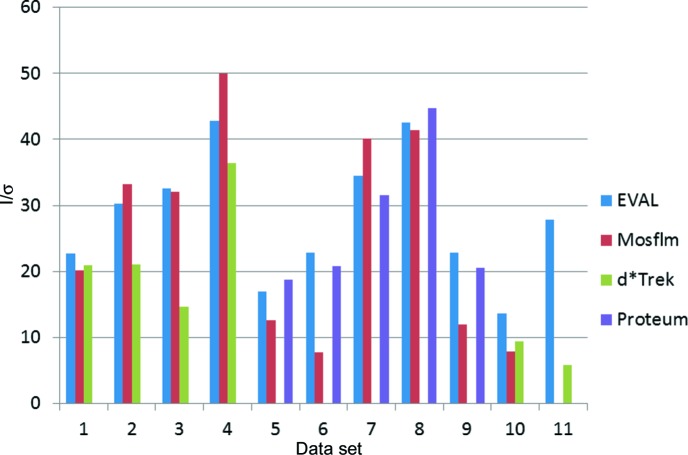
Merged *I*/σ values for data sets. 1: 4dd0; 2: 4dd2; 3: 4dd3; 4: 4dd9; 5: 4dd1; 6: 4dd4; 7:4dd6; 8: 4dd7; 9: 4ddc; 10: 4dda; 11:4ddb.

*I*/σ values for merged data can be found in Tables 2 ▶ and 3 ▶, and are summarized in Fig. 4 ▶. For the Rigaku data, the numbers for *EVAL* and *Mosflm* are in reasonable agreement, while d*Trek produces in general lower values, sometimes markedly so (4dd3, 4ddb). For the Bruker data, *EVAL* and the *PROTEUM2* (*SAINT* Version 7.68a) processing software are in close agreement even for crystals 5 and 9, which are in fact orthorhombic, leading to a lower redundancy for *EVAL* (Table 3 ▶). *Mosflm* has lower *I*/σ values in a majority of cases. The multiple-scan data collection with different detector positions may not be ideal for *Mosflm* without skilled fine tuning, *i.e.* which might be possible for the *MOSFLM* developers themselves. The accessibility of the raw diffraction data images linked to this article thus shows up an immediate advantage of archiving the raw diffraction data relating to a published article.

**Figure 5:**
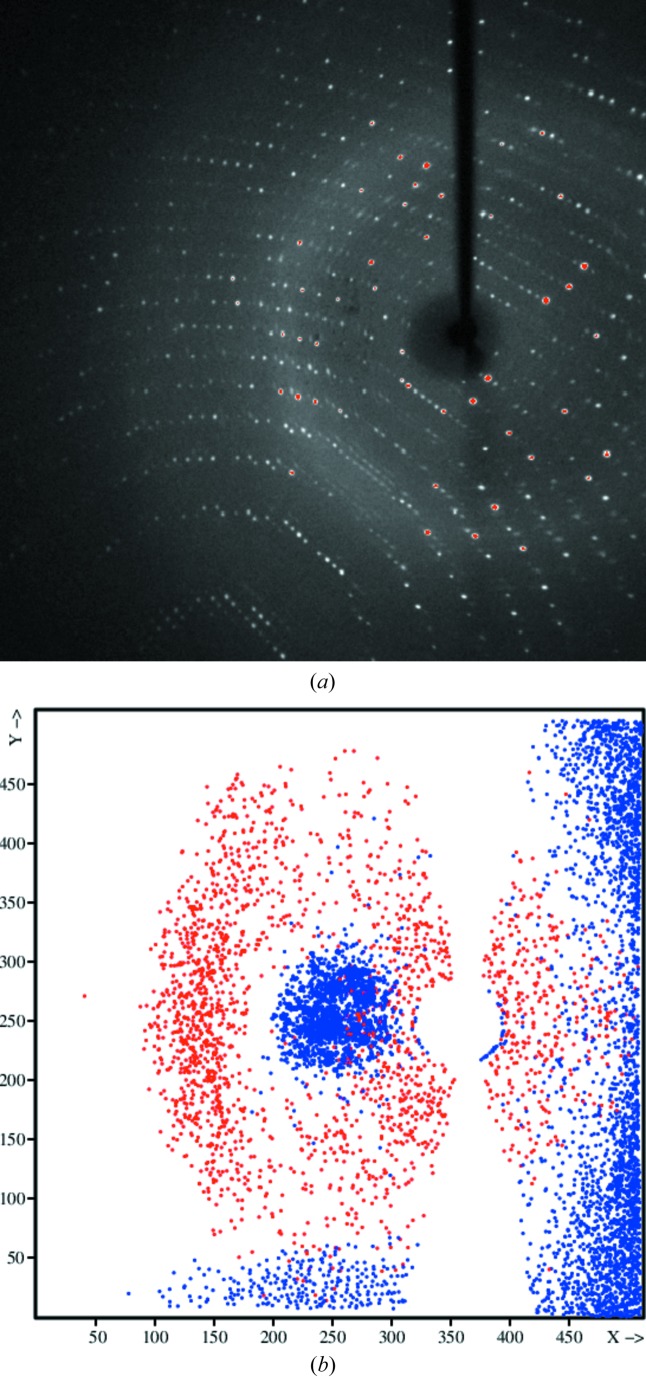
(*a*) Bruker diffraction data image of crystal 8. In the central part, left of the backstop shadow towards the solvent ring, the X-ray background scattering is seemingly lower, as shown in the dark stain-like area. (*b*) Reflections, projected onto the detector, for which the intensity deviates more than 4σ from the expected value.

Bruker diffraction data images are corrected for the relative sensitivity across the face of the detector by a flood-field image, determined with an isotropically scattering fluorescent sample. Careful inspection of the diffraction data images shows that some moisture had built up (dark variation in the background in the central part of the image) between the front protective screen and the phosphor layer of the detector (see Fig. 5 ▶ *a*). A projection of reflections rejected by *SADABS* onto the detector (4dd7; Fig. 5 ▶ *b*) shows that many occur in these areas of moisture, indicating systematic problems with the flood-field correction now not being appropriate as it would have been measured before the moisture build up. This problem can only be removed by a maintenance technician.

**Figure 6:**
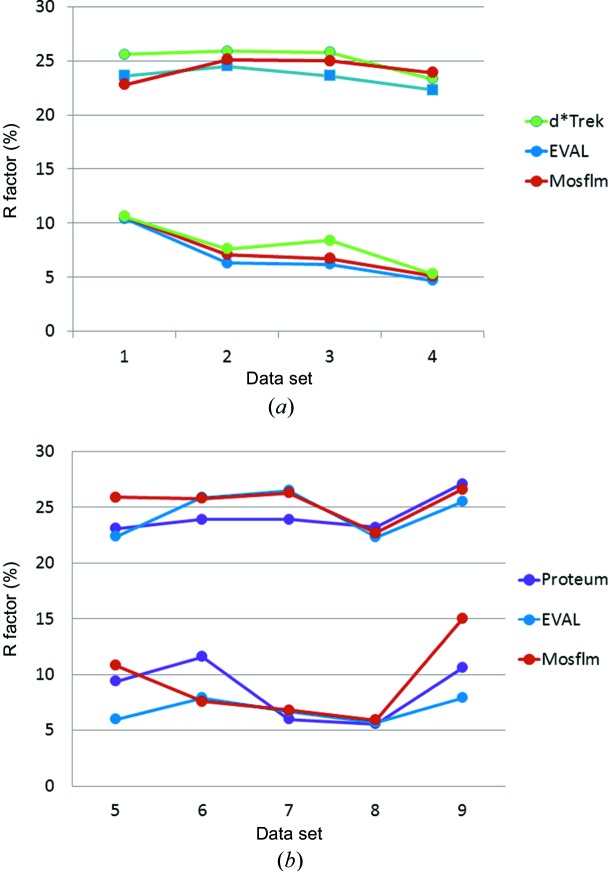
*R*_free_ (upper) and *R*_merge_ (lower) in % for crystals diffracting to 1.7 Å. (*a*) Rigaku data of crystals 1–4. (*b*) Bruker data of crystals 5–9.

For protein-model-refined crystal structures the *R* factors are often in the range 20–25%, while the intrinsic measurement errors are around 5%. Vitkup *et al.* (2002 ▶) show that the major contributions to this gap between *R* factors and the measurement errors are caused by the lack of a proper description of anisotropic protein motions, which can often not be determined because of the limited resolution of the data. At atomic diffraction resolution, spherical atomic scattering factors are a further inadequate approximation. Indeed *R*_merge_ values of our diffraction data sets range from 5 to 15% for the 1.7 Å crystals, while the *R*_free_ protein model refinement values are 22.3–26.3 (see Fig. 6 ▶). For the Rigaku diffraction data all the *R* factors agree closely between the data sets. For the Bruker diffraction data sets there is more spread. However the basic *R*_merge_/*R*_free_ gap is the same for each. Again crystals 5 and 9 are exceptions because of their orthorhombic symmetry.

#### 3.1.5. Crystal scattering power *versus* diffraction resolution   

**Figure 7:**
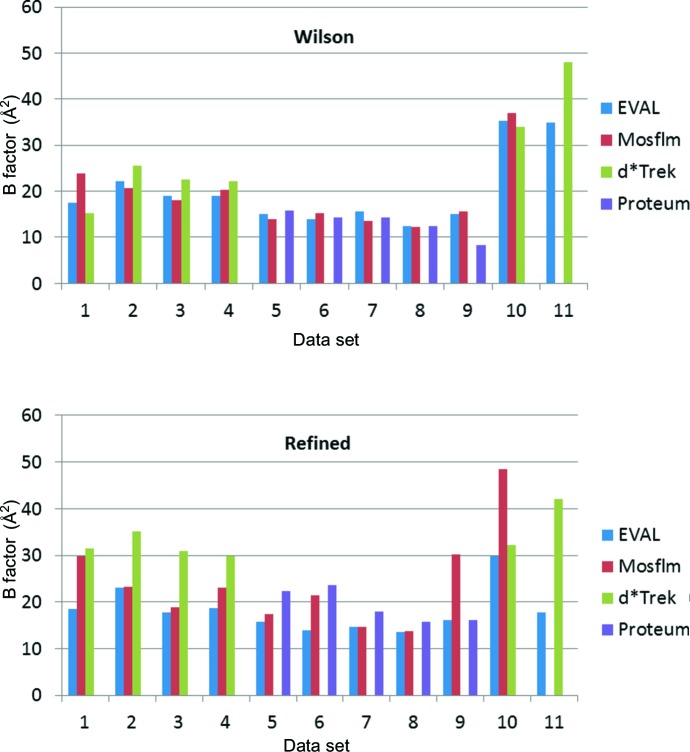
Wilson diffraction data *B* factors (marked ‘Wilson’) and protein-model-refined average isotropic atomic *B* factors (marked ‘refined’) (Å^2^) for all 11 crystals. The last two crystals diffract to a lower, *i.e.* poorer, resolution. 1: 4dd0; 2: 4dd2; 3: 4dd3; 4: 4dd9; 5: 4dd1; 6: 4dd4; 7: 4dd6; 8: 4dd7; 9: 4ddc; 10: 4dda; 11: 4ddb.

The incentive to start this work was an apparent systematic deviation between protein-model-refined *B* factors obtained with the diffraction data from different instruments and/or processing software. Thus we undertook data processing of all 11 data sets with the single software package *EVAL*. *EVAL*’s diffraction data processing statistics in Tables 2 ▶ and 3 ▶ show that the quality of the crystals varies somewhat, but mostly they diffract to approximately 1.7 Å, except for two crystals (4dda and 4ddb), which simply did not diffract further than 2.4–2.5 Å. In the highest diffraction resolution shells the average unmerged *I*/σ(*I*) varies between 0.9 and 3.5. Despite the difference in beam flux, detector quantum gain and measurement times, average individual *I*/σ(*I*) values are not grossly different (4.5–11.0; data not shown). Crystals that diffract to 1.7 Å were determined to have similar Wilson *B* factors for each diffractometer (Fig. 7 ▶), but clearly those from the Rigaku-processed diffraction data (crystals 1–4) are significantly larger than those from the Bruker-processed diffraction data (crystals 5–9). However, the difference is much smaller than the average atomic *B* factors from the protein model refinements (see §[Sec sec3.2]3.2 for a discussion on software for the protein model refinements). We can think of two reasons why this diffractometer hardware difference arises. First, Bruker diffraction data images are corrected for non-uniformity by flood-field images. Correction factors can be as much as 10–20%. Any errors in this correction procedure could have a systematic effect on the drop of intensity with 2θ and thus explicitly on the atomic *B* factors. However, reflection intensities in the Bruker diffraction data are measured at completely different positions on the detector, because of the various swing angles constituting a complete data set, so that such systematic effects are not likely. Secondly, high-order reflections have higher incidence angles in the case of Rigaku imaging plate data, with the detector set in the usual 2θ = 0 detector position. If the reflections are measured in the thin-phosphor regime (Chupas *et al.*, 2003 ▶), the X-ray absorption is proportional to the path length through the phosphor and intensities should be corrected (Zaleski *et al.*, 1998 ▶). However, generally, image plates are designed such that, for wavelengths larger than 1 Å, reflected X-ray beams are fully absorbed and such a correction would not be necessary. Still this effect could leave traces that will eventually end up in the protein model atomic *B* factors.

### 3.2. Comparison of diffraction data processing software   

All the crystal structures were solved using molecular replacement with *Phaser* (McCoy *et al.*, 2007 ▶) and restrained refinement with TLS (translation–libration–screw motion) in *REFMAC5* (Vagin & Teplyakov, 2010 ▶) in *CCP4i* (http://www.ccp4.ac.uk/ccp4i_main.php), using the lysozyme structure 2w1y as the molecular search model (Cianci *et al.*, 2008 ▶). Model building, adjustment and refinement were carried out using the *Coot* (Emsley & Cowtan, 2004 ▶) molecular-graphics program and *REFMAC5* in *CCP4i*, respectively. Metal ligand binding occupancies and their *B* factors were finally calculated using *SHELXTL* (Sheldrick, 2008 ▶).

#### 3.2.1. *B* factors   

**Figure 8:**
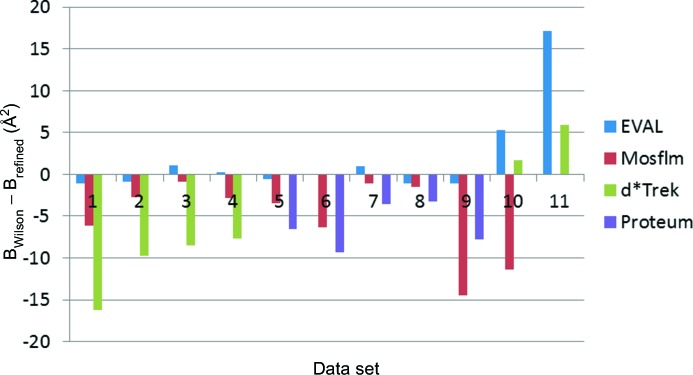
*B*_Wilson_ − *B*_refined_ (Å^2^) for the 11 crystals. 1: 4dd0; 2: 4dd2; 3: 4dd3; 4: 4dd9; 5: 4dd1; 6: 4dd4; 7: 4dd6; 8: 4dd7; 9: 4ddc; 10: 4dda; 11: 4ddb.

The Wilson *B* factors for the processed diffraction data of *EVAL* and *Mosflm* agree the closest. In general the agreement between the Wilson and protein-model-refined *B* factors is very good for *EVAL* and is somewhat less so for *Mosflm* (Fig. 8 ▶). One would expect a rough correspondence between Wilson *B* factor and the refined average individual atomic *B* factors, though the latter tend to be higher in general. Indeed, most numbers in Fig. 8 ▶ are negative, but the deviation is significantly larger for d*Trek. It appears that the diffraction data processing software may be critical to the published atomic displacement parameters of (protein) structures. Some caution has to be taken here as expert users of *Mosflm*, d*Trek and *PROTEUM* could find slightly different results. Some specific deviations have an easily explainable cause. For example, crystals 5 (4dd1) and 9 (4ddc) have significantly higher refined *B* factors with *PROTEUM* and *Mosflm* data processing, because loss of tetragonal symmetry was not recognized (see below), in our hands (JRH and ST), and therefore the difference in conformation between the two independent molecules was modelled as a type of static disorder. Data sets 10 (4dda) and 11 (4ddb) are exceptions because of the low resolution and concomitant TLS refinement only. Therefore the Wilson and refined *B* factors are not comparable. Wrongly estimated low-order reflection intensities may lead to, most obviously, erroneous Wilson *B* factors but also atomic *B* factors. *EVAL* rejected the lowest resolution reflections that were partly shadowed by the beam stop.

We analysed if significant differences could be found between the final refined structures or the initial electron density maps from which Pt atoms were located. The r.m.s. deviations between atom positions were in the range 0.1–0.6 Å for all pairwise software comparisons, except for crystal 1 where the d*Trek-refined structure deviated from the results from both *EVAL* and *Mosflm* by 0.5–1.0 Å. *B*-factor variations between residues were similar for all data, and d*Trek always has higher values. Apparently, the larger *B* factor for d*Trek is isotropic, *i.e.* it does not affect one part of the molecule more than others. Densities in 2*F*_o_–*F*_c_ or *F*_o_–*F*_c_ maps at Pt positions are similar in all cases.

The use of *EVAL* for processing all of the diffraction data sets provided a consistent platform for our large ensemble of data sets for the various protein and platinum compound model refinements with *REFMAC5* and then *SHELXTL*. Platinum occupancy values, and their standard deviations, were finally calculated using the results from three different diffraction data processing programs. We found that the differences in *B* factors do not impinge on the occupancies of Pt in cisplatin and carboplatin bound to lysozyme (Tanley *et al.*, 2012 ▶) as these agree to within ∼±5%.

#### 3.2.2. Contamination with ice   

**Figure 9:**
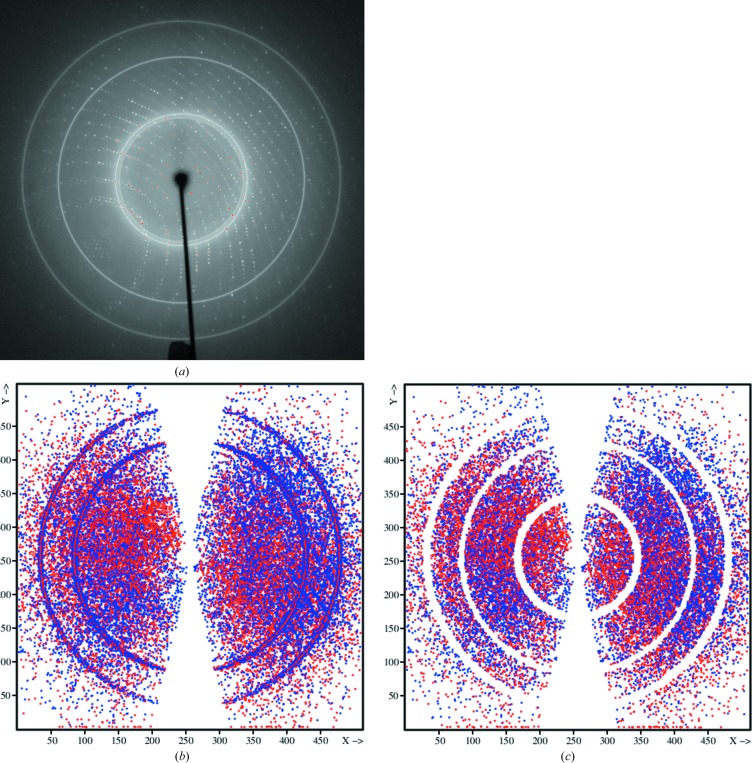
Diffraction pattern (*a*) and reflections deviating more than 3σ from the average of equivalents in *SADABS* for crystal 2 (4dd2), for both (*b*) untreated and (*c*) de-iced data.

Despite the use of cryoprotectants, some diffraction patterns indicate different levels of ice formation. It was uncertain what influence ice-contaminated reflection intensities would have in the protein model refinements. Diffraction data processing software sometimes has the option to avoid reflection integrations in suspect regions. In *EVAL* we decided to integrate all data and to decide afterwards which resolution regions we might want to discard. A diffraction data image of crystal 2 (4dd2) and projection of reflections with |*I* − 〈*I*〉|/σ > 4 by *SADABS* (Fig. 9 ▶) show that most of the reflections in ice regions will be rejected, so that no large problems were to be expected in the protein model refinement. Also *SADABS* rejections are shown after our ice-rejection procedure using *ANY* (Schreurs, 2007 ▶). The result is that for crystals 2 (4dd2), 10 (4dda), 11 (4ddb), 6 (4dd4) and 7 (4dd6) the completeness (Tables 2 ▶ and 3 ▶) dropped to 82–91%. The changes in *R*_merge_ were insignificant, whereas the protein model refinement *R*/*R*_free_ values were only noticeably different for the lower-resolution crystals: *R*/*R*_free_ = 21.4/29.9 *versus* 20.0/28.5 (de-iced) for crystal 10 (4dda) and 28.5/34.8 *versus* 21.4/27.9 for de-iced for crystal 11 (4ddb). It would seem that diffraction data scaling programs cannot reject such reflection groups if all diffraction equivalents are equally affected by ice scattering. This, however, is rarely the case and if the diffraction data redundancy is sufficiently large the reflections can actually be rejected, as done by *SADABS*.

#### 3.2.3. Loss of tetragonal symmetry cases   

**Figure 10:**
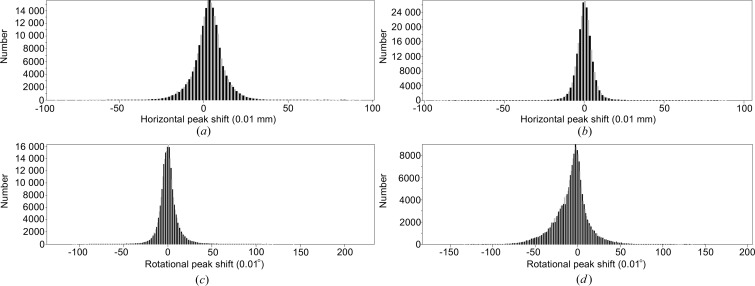
Distribution of horizontal (in units of 0.01 mm) and rotational (in units of 0.01°) peak shifts of data 4ddc in *EVAL15*. Horizontal peak shifts using (*a*) a tetragonal or (*b*) an orthorhombic unit cell; rotational peak shifts using (*c*) a tetragonal or (*d*) an orthorhombic unit cell.

In the *EVAL* software, *DIRAX* finds a first primitive lattice from peaks in a few diffraction images. Our experience is that when a large number of diffraction peaks are chosen the unit cell is sufficiently accurate for integration in *EVAL15* without a major difference between observed and predicted peak positions. *EVAL15* shifts the diffraction peaks to optimal positions for the profile fit as determined from χ^2^. If we are pleased with the agreement we usually do a post refinement to determine the best crystal unit cell for structure determination. If the errors are too large we may refine the unit cell (and orientation), restart the generation of box files at predicted positions and subsequently reintegrate with *EVAL15*. This may occur, for example, if goniometer offsets or detector positions are not known or not trusted. As mentioned in the *Metadata* [Sec sec3.1.2] section, we ignore these offsets and refine them with *EVAL*. However, for two crystals the errors were larger than what we are used to and larger than for the other crystals. Crystal 9 (4ddc), when indexed with 4/*mmm* symmetry, gave positional errors on the detector of 0.18 mm (corresponding to more than 2 pixels) and a 0.14° error in rotation angle, far too large in our view. In addition the ‘rlaue’ instruction in *ANY* gives *R*_merge_/*R*_meas_/*R*_pim_ of 0.176/0.180/0.038 and 0.127/0.130/0.028 for 4/*mmm* and *mmm*, respectively; *mmm* symmetry class was clearly an improvement. We were in fact warned because *DIRAX* persistently found significantly different dimensions for the *a*- and *b*-axis values, with whatever peaks we offered. Of course one can be tempted to assume the well known tetragonal symmetry of HEWL. Release of the constraint between *a* and *b* values in *PEAKREF* and subsequent integration with *EVAL15* solved the problem, leading to *a* = 77.94 and *b* = 79.09 Å instead of 78.52 Å, and the diffraction peak agreement became accurate to within 0.07 mm and 0.07° (Fig. 10 ▶). Close inspection showed that a similar phenomenon occurred with crystal 5 (4dd1).

## 4. Conclusions   

This joint project needed the network transfer of 35.3 Gb of raw diffraction data images between Manchester and Utrecht. As soon as the images arrived in Utrecht they were compressed to 20 Gb, using the *ncompress* lossless data compression package, to save disk space and because *EVAL* can read compressed images. It would have been efficient to compress before file transmission, but d*Trek, *PROTEUM* and *Mosflm* only process uncompressed images and therefore they were left untouched in Manchester. It took about 30 h of total transfer time to get the data across. As this was done one data set at a time, constrained by a typical working day, the transfer was spread over several days. In future it may be advisable to use on-the-fly compression (*e.g.* scp -C in Linux) during file transfer as well as a simple concatenation of the various data sets.

There exists long-term interest in performing comparative studies of hardware and software as exemplified by Helliwell *et al.* (1981 ▶). The present paper, one of the first to be accompanied with archiving of the raw data, may be the start of further comparative studies.

Comparison of diffractometer hardware was achieved by using *EVAL* processing. The Rigaku diffraction data sets have larger positional errors when compared with the Bruker diffraction data sets, which could be due to the crystal not being very well fixed into position, possibly as a result of vibrating instrument parts. The hardware is also partly responsible for the difference in Wilson *B* factors.

In comparing the software programs, the Wilson *B* factors are often significantly larger for the Rigaku data sets compared to the Bruker data sets, with *Mosflm* and *EVAL* agreeing closely for all 11 data sets

Also the refined atomic *B* factors were often significantly larger for d*Trek. This would mean that the data processing software may be critical to the published atomic displacement parameters of such protein structures. *Mosflm* performed worse in the processing of multiple-scan data with different detector positions, as compared with the single-detector-position Rigaku data. Despite differences in *B* factors of the refined structures derived from data of different processing software, the Pt occupancies were within a σ range of ±5% (see Tanley *et al.*, 2012 ▶). The availability of the raw diffraction images allows for independent assessment of software packages. The results described here may be biased in favour of *EVAL*, because we are the experts in this software.

In *EVAL* we implemented a procedure to avoid reflections affected by ice scattering. However, the results are not much different when no special care was taken, at least in these reasonably highly redundant diffraction data sets. We found that *SADABS* is capable of rejecting ice-affected reflections when the data have sufficiently high redundancy.

Without prior knowledge we would not have been able to discern sufficient metadata to carry out data processing of both types of diffraction images. This raises concerns with respect to long-term archiving of raw diffraction data. Care has to be taken that in the future unambiguous information is available, although this paper in itself is already a step towards providing the research community with knowledge of such metadata. The raw data will be deposited at Manchester University in 2013, so that software developers are able if they wish to improve on our data processing. Currently, a temporary depository is available at http://rawdata.chem.uu.nl.

Processed and derived data have been deposited with the PDB [PDB codes 3txb, 3txd, 3txe, 3txf, 3txg, 3txh, 3txi, 3txj and 3txk (new to this paper); PDB codes 4dd0, 4dd1, 4dd2, 4dd3, 4dd4, 4dd6, 4dd7, 4dd9, 4dda, 4ddb, 4ddc (from Tanley *et al.*, 2012 ▶)].

## Supplementary Material

Raw data: archive at Utrecht University containing images measured at Manchester University URL: http://rawdata.chem.uu.nl/#0001

Raw data: mirror of the raw data from Tardis at Monash University URL: http://vera183.its.monash.edu.au/protein_cisplatin_carboplatin

## Figures and Tables

**Table 1:** Data collection strategies (from Tanley *et al.*, 2012 ▶)

	Detector distance (mm)	Swing ()	Sweep ()	Rotation/frame ()	Generator filament current (mA)
Rigaku R-AXIS IV					
4dd0 (3txb)	100	0	360	1	20
4dd2 (3txd)	120	0	360	1	20
4dd3 (3txe)	120	0	272	1	20
4dd9 (3txi)	120	0	361	1	20
4dda (3txj)	135	0	181	1	20
4ddb (3txk)	200	0	360	1	20

Bruker PLATINUM^135^					
4dd1	60†	20	307.0	0.5	60
4dd4 (3txf)	50†	15.5	202.0	0.5	60
		8.0	38.0		
		12.0	35.0		
		22.0	52.0		
		22.0	63.0		
4dd6 (3txg)	60†	20	360.0	0.5	60
		20	360.0		
4dd7 (3txh)	50†	0	31.0	0.5	60
		0	180.0		
		0	360.5		
		20	360.5		
4ddc	50†	0	360.0	0.5	60
		20	360.0		

† Distance from face of the detector to the phosphor plane is an additional 6.6mm.

**Table:** Numbers in brackets are the ‘outer resolution shell’ values.

Crystal	1			2			3		
PDB code	3txb	4dd0		3txd	4dd2		3txe	4dd3	
Software	d*Trek	*EVAL*	*Mosflm*	d*Trek	*EVAL*	*Mosflm*	d*Trek	*EVAL*	*Mosflm*
Unit cell†	*a* = 78.66	*a* = 78.69	*a* = 78.61	*a* = 78.88	*a* = 78.91	*a* = 78.90	*a* = 78.66	*a* = 78.53	*a* = 78.54
	*c* = 36.96	*c* = 36.90	*c* = 36.91	*c* = 36.99	*c* = 36.99	*c* = 37.00	*c* = 37.44	*c* = 37.36	*c* = 37.38
Observed reflections	416806	336926	390544	366668	271407	267042	248915	239297	243139
Unique reflections	17504	13312	14988	16526	14462	10457	13378	13144	12223
Resolution ()	55.621.59 (1.641.59)	19.671.70 (1.761.70)	18.771.63 (1.731.63)	55.771.53 (1.591.53)	19.731.55 (1.601.55)	26.991.78 (1.871.78)	55.511.70 (1.761.70)	31.011.70 (1.761.70)	17.711.75 (1.841.75)
Completeness (%)	99.3 (93.2)	99.9 (100)	99.9 (100)	91.4 (38.4)	82.6‡ (53.0)	88.4 (100)	98.0 (82.3)	98.1 (82.9)	99.5 (95.8)
*R*_merge_	0.106 (0.377)	0.104 (0.64)	0.106 (1.36)	0.076 (0.327)	0.063 (0.456)	0.071 (0.24)	0.084 (0.395)	0.062 (0.314)	0.067 (0.30)
Merged mean *I*/	20.9 (8.4)	22.7 (4.4)	20.1 (2.2)	21.1 (2.1)	30.3 (2.1)	33.2 (11.1)	14.7 (4.2)	32.6 (7.3)	32.0 (8.9)
Redundancy	25.8 (18.9)	25.3 (25.8)	26.1 (24.6)	11.9 (2.5)	18.8 (4.2)	25.5 (24.8)	18.9 (14.0)	18.3 (13.2)	19.9 (18.3)
*B* Wilson	15.2	17.4	23.8	25.5	22.1	20.6	22.5	18.9	18.0
Average atomic *B* factor ()	31.4	18.5	29.9	35.2	23.0	23.3	31.0	17.8	18.9
*R* factor/*R*_free_ (%)	20.9/25.6	18.7/23.6	17.7/22.8	19.8/25.9	20.0/24.5	18.9/25.1	20.0/25.8	19.2/23.6	18.9/25.0
*R* factor (all)	20.9	18.9	18.0	20.6	20.2	19.2	21.6	19.4	19.2
R.m.s.d. bond lengths ()/angles ()	0.0359/2.4021	0.017/1.924	0.0192/1.8505	0.0259/2.0605	0.0187/2.0721	0.0188/1.8168	0.0241/1.9858	0.0191/1.9181	0.0195/1.8769

**Table:** 

Crystal	4			10			11	
PDB code	3txi	4dd9		3txj	4dda		3txk	4ddb
Software	d*Trek	*EVAL*	*Mosflm*	d*Trek	*EVAL*	*Mosflm*	d*Trek	*EVAL*
Unit cell†	*a* = 78.66	*a* = 78.53	*a* = 78.04	*a* = 78.53	*a* = 78.37	*a* = 78.48	*a* = 79.46	*a* = 79.64
	*c* = 36.98	*c* = 37.36	*c* = 37.98	*c* = 36.72	*c* = 36.58	*c* = 36.99	*c* = 36.96	*c* = 37.02
Observed reflections	358383	296297	310742	56396	49543	61525	13178	79530
Unique reflections	15336	15451	11554	4343	4120	4991	4150	3234
Resolution ()	55.181.60 (1.661.60)	31.011.70 (1.761.70)	26.841.78 (1.881.78)	55.532.48 (2.572.48)	19.592.40 (2.482.40)	25.462.38 (2.512.38)	56.193.00 (3.113.00)	30.942.50 (2.582.50)
Completeness (%)	98.8 (90.5)	98.9 (89.7)	100 (100)	99.2 (93.1)	85.3‡ (100)	99.9 (100)	91.3‡ (91.9)	72 (100)
*R*_merge_	0.053 (0.220)	0.047 (0.154)	0.051 (0.13)	0.199 (0.412)	0.147 (0.607)	0.226 (0.87)	0.15 (0.266)	0.136 (0.528)
Merged mean *I*/	36.4 (6.2)	42.8 (6.6)	50.0 (22.7)	9.4 (4.8)	13.6 (4.0)	7.8 (2.5)	5.8 (3.2)	27.8 (6.3)
Redundancy	23.7 (7.4)	19.2 (4.5)	26.9 (25.8)	12.9 (11.5)	12.1 (12.8)	12.3 (12.7)	3.18 (3.15)	24.6 (24.5)
*B* Wilson	22.2	18.9	20.3	34.0	35.2	37.0	48.0	34.9
Average atomic *B* factor ()	29.9	18.6	23.1	32.3	29.9	48.4	42.1	17.8
*R* factor/*R*_free_ (%)	18.7/23.3	18.3/22.3	18.9/23.9	22.3/28.9	20.0/28.5	21.2/26.6	21.1/25.8	21.4/27.9
*R* factor (all)	19.1	18.5	19.2	22.5	20.4	21.4	20.2	21.6
R.m.s.d. bond lengths ()/angles ()	0.0280/2.3712	0.0200/2.0684	0.0199/2.0314	0.0219/1.94328	0.0126/2.3771	0.0186/1.6260	0.0217/2.0624	0.007/1.173

Processing with *Mosflm* did not succeed.

† Space group is *P*4_3_2_1_2 for all data.

‡ Reflections contaminated with ice scattering were removed from the data using a de-ice procedure.

**Table:** Numbers in brackets are the ‘outer resolution shell’ values.

Crystal	5			6			7		
PDB code		4dd1		3txf	4dd4		3txg	4dd6	
Software	*PROTEUM2*	*EVAL*	*Mosflm*	*PROTEUM2*	*EVAL*	*Mosflm*	*PROTEUM2*	*EVAL*	*Mosflm*
Unit cell†	*a* = 78.78	*a* = 78.88	*a* = 78.72	*a* = 78.44	*a* = 78.83	*a* = 79.11	*a* = 78.08	*a* = 78.01	*a* = 78.05
	*c* = 37.28	*b* = 78.70	*c* = 37.29	*c* = 36.97	*c* = 37.02	*c* = 37.06	*c* = 37.11	*c* = 37.07	*c* = 37.08
		*c* = 37.07							
Observed reflections	118456	131592	87575	176370	173061	173696	320101	272733	248797
Unique reflections	11366	25216	9476	13407	11859	18753	13147	10901	9458
Resolution ()	35.231.80 (1.891.80)	18.041.70 (1.761.70)	19.091.83 (1.931.83)	55.471.69 (1.791.69)	18.921.70 (1.761.70)	17.63 1.52 (1.621.52)	37.101.70 (1.801.70)	18.921.70 (1.761.70)	18.931.83 (1.931.83)
Completeness (%)	99.9 (100)	97.7 (92.2)	86.9 (79.3)	95.9 (75.2)	88.6‡ (100)	69.11 (83.5)	99.6 (97.5)	82.8‡ (100)	88.5 (77.4)
*R*_merge_	0.094 (0.278)	0.06 (0.200)	0.108 (0.28)	0.116 (0.357)	0.079 (0.313)	0.076 (1.33)	0.060 (0.286)	0.067 (0.306)	0.068 (0.22)
Merged mean *I*/	18.8 (5.71)	16.9 (4.97)	12.6 (4.2)	20.8 (7.82)	22.8 (4.8)	7.7 (0.60)	31.5 (5.48)	34.5 (8.0)	40.1 (11.0)
Redundancy	10.4 (7.9)	5.4 (4.4)	9.24 (6.7)	13.1 (5.9)	14.6 (9.1)	9.26 (3.2)	24.3 (16.2)	25.0 (18.0)	26.3 (19.1)
*B* Wilson	15.8	15.1	13.9	14.3	14.0	15.2	14.3	15.7	13.5
Average atomic *B* factor ()	22.3	15.7	17.4	23.6	14.0	21.5	17.9	14.7	14.6
*R* factor/*R*_free_ (%)	17.7/23.1	18.8/22.4	19.6/25.9	17.9/23.9	20.2/25.9	22.1/25.8	18.1/23.9	21.4/26.5	19.5/26.3
*R* factor (all)	18.1	19.1	19.9	20.8	20.5	22.3	18.5	21.6	19.8
R.m.s.d. bond lengths ()/angles ()	0.0267/2.1128	0.020/2.043	0.0191/3.3062	0.0207/2.1302	0.018/1.861	0.0221/1.7157	0.0261/2.2494	0.0183/2.134	0.0176/2.1269

**Table:** 

Crystal	8			9		
PDB code	3txh	4dd7			4ddc	
Software	*PROTEUM2*	*EVAL*	*Mosflm*	*PROTEUM2*	*EVAL*	*Mosflm*
Unit cell†	*a* = 78.84	*a* = 78.82	*a* = 78.80	*a* = 78.60	*a* = 78.94	*a* = 78.49
	*c* = 37.03	*c* = 37.02	*c* = 37.00	*c* = 37.01	*b* = 79.08	*c* = 36.94
					*c* = 36.98	
Observed reflections	361272	500514	323705	30705	329619	209107
Unique reflections	13494	15970	12839	4377	21884	11716
Resolution ()	55.751.69 (1.791.69)	20.671.60 (1.651.60)	19.111.72 (1.821.72)	54.451.54 (2.372.25)	19.161.80 (1.861.80)	18.721.72 (1.821.72)
Completeness (%)	99.8 (99.4)	99.9 (99.9)	99.9 (100)	100 (100)	99.9 (100)	92.3 (99.1)
*R*_merge_	0.0557 (0.156)	0.057 (0.179)	0.059 (0.15)	0.106 (0.583)	0.079† (0.213)	0.15 (0.74)
Merged mean *I*/	44.8 (10.7)	42.5 (7.0)	41.4 (9.9)	20.53 (23.18)	22.9† (4.4)	12.0 (1.8)
Redundancy	26.7 (9.8)	31.4 (9.4)	25.2 (9.4)	19.8 (3.91)	15.1 (5.6)	17.8 (6.2)
*B* Wilson	12.2	12.5	12.2	8.4	15.1	15.7
Average atomic *B* factor ()	15.8	13.6	13.7	16.2	16.2	30.2
*R* factor/*R*_free_ (%)	16.7/23.2	18.3/22.3	17.0/22.7	18.1/27.1	21.8/25.5	20.1/29.0
*R* factor (all)	16.9	18.5	17.3	19.2	21.9	20.6
R.m.s.d. bond lengths ()/angles ()	0.0307/2.5650	0.0200/2.0684	0.0210/2.0594	0.0274/2.3062	0.0181/1.8160	0.0165/1.5159

The resolution was cut back because at higher resolutions the protein structure refinement gave poor *R*/*R*_free_ statistics.

† Space group is *P*4_3_2_1_2 for all data except for *EVAL* data 4dd1 and 4ddc, where it is *P*2_1_2_1_2_1_.

‡ Reflections contaminated with ice scattering were removed from the data using a de-ice software procedure.

**Table 4:** Average errors in reflection positions for EVAL

	4dd0†	4dd2†	4dd3†	4dd9†	4dda†	4ddb†	4dd1	4dd4‡	4dd6	4dd7	4ddc
Number	203324	258947	181630	267749	32094	44482	98670	137208	216924	431390	276914
Angular ()	0.096	0.103	0.175	0.157	0.104	0.051	0.047	0.089	0.025	0.029	0.072
Positional (mm)	0.168	0.216	0.366	0.321	0.244	0.176	0.053	0.086	0.028	0.028	0.070
Rotation ()	0.188	0.165	0.172	0.196	0.128	0.203	0.049	0.074	0.046	0.028	0.073

† Orientation of the unit-cell matrix was refined for each box file (1000 reflections) separately.

‡ Orientation of the unit-cell matrix was refined for each scan separately.
